# Clinical and Analytical Performance of the AP Biotech Discovery^®^ Dengue Virus RT-PCR Detection Kit

**DOI:** 10.3390/v18080914

**Published:** 2026-08-20

**Authors:** Valentina Hjelt, Agustina Dronseck, Aldana Magalí Schey, María Belén Martí, Luciano Civerchia, Ana Laura Cavatorta, Eduardo Codino, Virginia Nader, Natalia Andrea Garro, Federico Manuel Aranda, Lilia Mammana, Sergio Giamperetti, Clara Theaux, Anisa Marchissio, Valeria Estefanía Burani, Erica Natalia Luczak, María Martina Bellucci, Gabriela García, Florencia Cayrol, María Belén Bouzas, Sergio Grutadauria, Lucila Brocardo

**Affiliations:** 1 AP Biotech, Lomas de Zamora B1832FYA, Buenos Aires, Argentina; vhjelt@apbiotech.com.ar (V.H.); adronseck@apbiotech.com.ar (A.D.); aschey@apbiotech.com.ar (A.M.S.); 2 Laboratorio Mixto de Investigación Traslacional en Salud, Centro de Tecnología en Salud Pública (CTSP), Facultad de Ciencias Bioquímicas y Farmacéuticas, Universidad Nacional de Rosario, Rosario S2002LRK, Santa Fe, Argentinalciverchia76@gmail.com (L.C.); acavatorta@fbioyf.unr.edu.ar (A.L.C.);; 3 Laboratorio Central, Sanatorio Allende, Córdoba X5000BFB, Córdoba, Argentina; vnader@sanatorioallende.com (V.N.); nagarro@sanatorioallende.com (N.A.G.); sgrutadauria@sanatorioallende.com (S.G.); 4 Unidad de Virología, División Análisis Clínicos, Hospital de Infecciosas F. J. Muñiz, Ciudad Autónoma de Buenos Aires C1282AEN, Argentina; federicoaranda.04@gmail.com (F.M.A.); lmammana@buenosaires.gob.ar (L.M.); giamperetti@hotmail.com (S.G.); 5 Sector de Biología Molecular, División Laboratorio, Hospital General de Agudos Dr. Carlos G. Durand, Ciudad Autónoma de Buenos Aires C1405DCS, Argentina; clara_theaux@hotmail.com (C.T.); anisamarchissio@gmail.com (A.M.); 6 Laboratorio de Biología Molecular, Sanatorio Juncal, Temperley B1834GUK, Buenos Aires, Argentina; valeburani@gmail.com (V.E.B.); enluczak@gmail.com (E.N.L.); 7 Laboratorio de Virología, Hospital Interzonal General de Agudos Dr. Pedro Fiorito, Avellaneda B1870ARG, Buenos Aires, Argentina; mariamartinabellucci@gmail.com (M.M.B.); pequegabygarcia@hotmail.com (G.G.); 8 Laboratorio de Oncohematología Experimental y Traslacional, Instituto de Investigaciones Biomédicas (BIOMED, CONICET-UCA), Ciudad Autónoma de Buenos Aires C1107AFF, Argentina; florencia_cayrol@uca.edu.ar; 9 Jefatura de División de Análisis Clínicos, Hospital de Infecciosas F. J. Muñiz, Ciudad Autónoma de Buenos Aires C1282AEN, Argentina; mariabbouzas@gmail.com

**Keywords:** dengue, real-time RT-PCR, in vitro diagnostics (IVD), molecular diagnosis, limit of detection, specificity, clinical validation

## Abstract

Dengue is a mosquito-transmitted viral disease caused by the positive-sense RNA dengue virus (DENV), which comprises four antigenically distinct serotypes (1–4). Although DENV circulates predominantly in tropical and subtropical regions, its global incidence continues to increase. As most patients present viremia at the onset of clinical manifestations, real-time RT-PCR has become a widely adopted method for early dengue detection. The AP Biotech Discovery^®^ Dengue Virus RT-PCR Detection Kit enables qualitative detection of DENV RNA (serotypes 1–4) by real-time RT-PCR in serum or plasma samples. The assay was recently cleared by ANMAT (National Administration of Drugs, Food and Medical Technology) as an in vitro diagnostic (IVD) assay. The Limit of Detection (LoD) was calculated for all 4 serotypes. Clinical validation was conducted among 296 serum and 221 plasma samples previously characterized as DENV-positive and -negative specimens. Among the positive samples, 56 had serotype characterization, including 25 DENV-1 and 31 DENV-2 samples. The resulting clinical sensitivity and specificity were 97.8%, 100.0% and 100.0%, 99.1% respectively. Additional validation studies demonstrated high reproducibility across three independent laboratories, absence of cross-reactivity with other febrile pathogens, and no interference from endogenous and exogenous substances at clinical levels. Collectively, these findings support the AP Biotech Discovery^®^ Dengue Virus RT-PCR Detection Kit (AP Biotech, Lomas de Zamora, Argentina) as a sensitive, specific, and highly reproducible device for early dengue diagnosis and surveillance.

## 1. Introduction

Dengue is an infectious disease caused by dengue virus (DENV), a member of the *Flavivirus* genus. DENV contains a positive-sense single-stranded RNA genome of approximately 11 kb, and four antigenically distinct serotypes have been identified (DENV1, DENV2, DENV3, and DENV4) [1]. Transmission is mediated primarily by the bite of Aedes mosquitoes, which serve as competent vectors of DENV. These mosquitoes are widely distributed across tropical and subtropical regions worldwide [2,3].

Dengue virus is rapidly expanding its geographical footprint, becoming endemic in more than 100 countries [4,5], and placing nearly half of the world’s population at risk of infection [6]. In the Americas, dengue represents the most prevalent arboviral disease, with outbreaks historically occurring every three to five years [7,8,9]. However, climate change and rapid urbanization have contributed to increasing the incidence and to greater co-circulation and diversity of DENV serotypes. The 2024 season was unprecedented in Latin America and the Caribbean, which recorded nearly 13 million suspected cases—a 361% increase relative to the previous five-year average [10,11]. Argentina experienced its largest dengue epidemic to date, with reported cases increasing from approximately 150,000 in 2023 to nearly half a million during the first months of 2024, along with more than 300 associated deaths [12]. This outbreak differed from previous seasons not only in magnitude but also in geographical expansion and serotype predominance with DENV2 being the most frequently detected [13,14]. Although the precise global burden remains a subject of active surveillance, these trends pose a critical threat to public health and the global economy [15,16,17], with annual costs in major regional economies, such as Brazil, reaching USD 1.2 billion [18].

The true magnitude of dengue cases is likely underestimated because many infections are asymptomatic or mildly symptomatic and therefore remain undiagnosed or unreported [19,20]. Symptomatic dengue disease may present as an acute febrile illness with nonspecific manifestations such as fever, headache, myalgia, and arthralgia [1]. A minority of cases may progress to severe dengue with hemorrhage, organ impairment, or shock [21,22]. Because these clinical manifestations overlap with those caused by other arboviral and febrile infections, clinical assessment alone is insufficient to identify the etiological agent.

Accurate laboratory diagnosis is therefore essential for patient management, epidemiological surveillance, and outbreak response. Direct detection of viral components is the most informative within the first six days after symptoms onset. Real-time reverse transcription polymerase chain reaction (RT-PCR) is the most sensitive method for diagnosing acute DENV infection through the detection of viral RNA in serum or plasma samples. Here, we describe the analytical and clinical validation of the AP Biotech Discovery^®^ Dengue Virus RT-PCR Detection Kit and its capability to detect all DENV serotypes.

## 2. Materials and Methods

### 2.1. Assay Design

The AP Biotech Discovery^®^ Dengue Real Time RT-PCR Detection Kit (AP Biotech, Lomas de Zamora, Argentina) was designed to detect DENV1, 2, 3, and 4 in serum or plasma samples and to be used in a laboratory environment, by qualified users as an in vitro diagnosis medical device (IVD). The test corresponds to nucleic acid amplification technology (NAAT). DENV detection relies on specific amplification of conserved genomic regions, NS5 (DENV1), the *E* gene (DENV2/4), and the *M* gene (DENV3), using specific primers and FAM-labeled probes, allowing the kit to accurately detect all clinically relevant serotypes, without serotype discrimination. The human *RNase P* gene, detected with a VIC-labeled probe, serves as an endogenous internal control (IC) to monitor sample quality, nucleic acid extraction, reverse transcription, and amplification. The assay incorporates a dUTP/Uracil-DNA glycosylase (UDG) system to prevent carry-over contamination.

The RT-PCR is assembled in multiplex format by combining only two ready-to-use reagents: PCR Reaction Mix and PCR Enzyme Mix. The kit also includes Positive and Negative Control, formulated for direct use to validate PCR runs. The real-time RT-PCR protocol was optimized to 70 min total and can be performed in regular real-time instruments, compatible with FAM and VIC fluorophore detection.

### 2.2. Surrogate Samples

Analytical performance of the AP Biotech Discovery^®^ Dengue Virus RT-PCR Detection Kit was assessed using surrogate samples prepared in a biological matrix, either spiked or not spiked with quantified analyte material. The DENV-negative biological matrix (eNCM) consisted of purified and extracted total nucleic acid (DNA/RNA) derived from pooled serum or plasma samples that tested negative for DENV using a commercial DENV detection PCR kit. Quantified materials of the four DENV serotypes derived from viral culture were provided by Guangzhou BDS Biological Technology Co., Ltd. (BDS, Guangzhou, China). To assess analytical specificity, a panel of non-target viral reference materials was sourced from the following suppliers: Vircell S.L. (Granada, Spain), Guangzhou BDS Biological Technology Co., Ltd. (BDS, China), and the American Type Culture Collection (ATCC, Manassas, VA, USA).

### 2.3. Molecular Biology Diagnosis Platform

AP Biotech Discovery^®^ Dengue Virus RT-PCR Detection Kit was validated as part of a diagnosis platform, integrating semi-automated nucleic acid extraction and purification and real-time RT-PCR, both instruments and reagents. Nucleic acids were extracted from clinical samples and quantified materials using the MagaBio Plus Virus DNA/RNA Purification Kit II with the GenePure Pro automated extractor (AP Biotech, Lomas de Zamora, Argentina), according to the manufacturer’s instructions. Briefly, 300 µL of each sample was processed, and nucleic acids were eluted in 60 µL of elution buffer. DENV RNA was detected using the AP Biotech Discovery^®^ Dengue Virus RT-PCR Detection Kit. Reactions were prepared with 19 μL of PCR Reaction Mix and 1 μL of PCR Enzyme Mix, followed by the addition of 5 μL of extracted nucleic acid (final volume 25 μL). Amplification was performed on a QuantGene 9600 system (AP Biotech, Lomas de Zamora, Argentina) under the following conditions: reverse transcription at 50 °C for 5 min; initial denaturation at 95 °C for 30 s; and 45 cycles of 95 °C for 5 s and 60 °C for 35 s. Runs were considered valid when the Positive Control showed a cycle threshold (Ct) ≤ 32 in both FAM and VIC channels and the Negative Control showed no amplification or Ct > 40. Samples were deemed invalid in the absence of internal control amplification. A result was interpreted as DENV-detectable when a typical sigmoidal amplification curve was observed in the FAM channel (Ct ≤ 40) with concurrent internal control amplification in the VIC channel (Ct ≤ 36).

### 2.4. Analytical Validation

#### 2.4.1. Analytical Sensitivity for DENV1-4

For PCR efficiency and linear behavior evaluation, spiked surrogate samples were prepared by serial dilutions of quantified DENV1 to DENV4 RNA, generating concentrations ranging from 2.0 × 10^5^ to 3.2 × 10^2^ copies/mL for DENV1 and DENV4 and from 2.0 × 10^5^ to 7.8 × 10^2^ copies/mL for DENV2 and DENV3. Six replicates were tested for each concentration level. DENV Ct values vs. concentration in copies/mL (in logarithmic scale) were plotted, and linear regression analysis was performed for each DENV serotype. PCR amplification efficiency (E) was calculated from the slope of the standard curve and was considered acceptable following standard criteria [23].

The limit of detection at 95% probability (LoD_95_) was established using two independent reagent batches. For each DENV serotype, RNA dilution series were tested in five concentrations per serotype, evaluated over three consecutive days, with six replicates per concentration, resulting in 54 replicates per reagent batch [24]. Statistical analysis was performed using MedCalc software version 23.4.2. The LoD_95_ was estimated by Probit regression analysis with a 95% confidence interval (CI), followed by logarithmic reversion.

#### 2.4.2. Cross-Reactivity

Pathogens were selected based on clinical relevance, including other arthropod-borne arboviruses, viruses detectable in serum or plasma, and pathogens associated with symptoms overlapping dengue disease [25]. High-quality quantitative reference materials were obtained for 15 non-target microorganisms, which were tested in vitro at clinically relevant high concentrations: Chikungunya virus (CHIKV) (1 × 10^5^ copies/mL), Cytomegalovirus (CMV) (1 × 10^5^ copies/mL), Epstein–Barr Virus (EBV) (1 × 10^5^ copies/mL), Hepatitis B Virus (HBV) (1 × 10^5^ copies/mL), Hepatitis C Virus (HCV) (1 × 10^5^ copies/mL), Herpes Simplex Virus type 1 (HSV-1) (1 × 10^5^ copies/mL), Herpes Simplex Virus type 2 (HSV-2) (5 × 10^4^ copies/mL), Human Immunodeficiency Virus type 1 (HIV-1) (1 × 10^4^ copies/mL), Influenza A H1N109 (Flu A) (1 × 10^5^ copies/mL), Influenza B (Flu B) (1 × 10^5^ copies/mL), St. Louis Encephalitis Virus (SLEV) (1 × 10^5^ copies/mL), Varicella-Zoster Virus (VZV) (1 × 10^5^ copies/mL), West Nile Virus (WNV) (1 × 10^5^ copies/mL), Yellow Fever Virus (YFV) (2.3 × 103 copies/mL), and Zika Virus (ZIKV) (1 × 10^5^ copies/mL). Surrogate samples were supplemented with purified quantified genomes from non-target microorganisms, assuming a 1:1 equivalence between genome copies/mL and PFU/mL. Each microorganism was tested in triplicate. Cross-reactivity was considered absent when no positive DENV signal was detected in any of the evaluated non-target samples.

#### 2.4.3. Interference Substances

Potential endogenous and exogenous interfering substances were evaluated in both the presence and absence of DENV RNA [26]. A total of eight interferents were assessed at clinically relevant concentrations [27]: bilirubin (475 µmol/L), human haemoglobin (2 g/L), human serum proteins (1 g/L), triglycerides (37 mmol/L), and cholesterol (3 mmol/L) (Molecular Depot); acetaminophen (100 µg/mL) and amoxicillin (7.5 mg/L) (Roemmers, Olivos, Buenos Aires, Argentina); and dexamethasone (5 µg/mL) (Sidus, Olivos, Buenos Aires, Argentina). Interference testing was performed testing 15 replicates of surrogate samples supplemented with the respective interfering substance and either spiked or not spiked with DENV RNA (tested concentration equivalent to 3 × LoD). The following equation was used to calculate the percentage of interference for Internal Control and DENV Ct values:

$$\%\;Interference=\;{(X}^{TS}-X^{CS})/X^{CS}$$

$X^{TS}$ = Mean Ct result obtained from the test sample containing the IS.

$X^{CS}$ = Mean Ct result obtained from the control sample without the IS.

No additional testing was required if the interference percentage fell within the acceptable range of ±10%.

#### 2.4.4. Precision

Intra-laboratory precision was evaluated in accordance with CLSI guideline EP05-A3 [28]. A panel of surrogate samples was prepared at three analyte concentrations: moderate positive (6 × LoD), low positive (3 × LoD), and negative (0.05 × LoD). Each concentration level was tested over five non-consecutive days by two independent operators using three reagent lots. Five replicates per sample were analyzed in each run, resulting in a total of 150 replicates per concentration level. The following criteria were applied: (a) a negative sample was expected to exhibit a DENV-negative result in more than 95% of replicates, (b) a low-positive sample was expected to show positive results in 95% to 100% of replicates; and (c) a moderately positive sample was expected to yield 98% to 100% of replicates as “DENV-positive”. Precision was determined using a three-way nested analysis of variance (ANOVA) to calculate standard deviations (SD) and coefficients of variation (CV%) of Ct values. Variability assessment included repeatability (within-run variability), day-to-day variability, operator, and reagent lot.

Statistical analyses were performed in Python 3.11.9 (Python Software Foundation), using the pandas library for data handling and statsmodels for ordinary least squares (OLS) modeling and ANOVA calculations [29]. For each concentration level, the observed agreement was calculated as the percentage of expected results (positive or negative, as appropriate) relative to the total number of replicates, and ninety-five percent confidence intervals (95% CI) were estimated using the Wilson score method [30,31].

#### 2.4.5. Reproducibility

Reproducibility was evaluated across three independent testing sites. The surrogate sample panel was identical to that used in the precision study. At each site, five replicates per sample were tested over five non-consecutive days using the same reagent lot, yielding a total of 75 replicates per concentration level across sites. Interlaboratory reproducibility was estimated using a two-way nested ANOVA to calculate SD and CV% of Ct values. Sources of variability included repeatability (within-run variability), day-to-day variability, and testing site. Observed agreement was calculated as described for precision analysis. Statistical analyses were also performed in Python 3.11.9 (Python Software Foundation).

### 2.5. Clinical Validation

#### 2.5.1. Clinical Performance

The clinical performance of the AP Biotech Discovery^®^ Dengue Virus RT-PCR Detection Kit was assessed through a retrospective, multicenter study and included a total of 296 serum and 221 plasma samples. Serum samples were provided by three public laboratories: Hospital de Enfermedades Infecciosas Francisco Javier Muñiz and Hospital General Carlos G. Durand (Buenos Aires, Argentina), Hospital Dr. Pedro Fiorito (Avellaneda, Argentina), and two private healthcare institutions—Sanatorio Juncal (Temperley, Argentina) and Sanatorio Allende (Córdoba, Argentina). Clinical validation for serum samples was performed in-house. Plasma samples were provided by Hospital General Carlos G. Durand and Sanatorio Allende (Córdoba, Argentina), where clinical validation assays were also performed. Samples were originally collected from patients with suspected dengue infection within the first six days of symptom onset during the 2023–2024 dengue outbreak in Argentina and tested according to each institution’s routine diagnostic protocol. From 182 DENV-positive serum samples, 56 had serotype characterization, resulting in the analysis of 25 DENV-1 and 31 DENV-2 samples during clinical validation. Residual volumes were stored at −70 °C until use. At the time of inclusion, all samples had been previously characterized for DENV RNA detection and were re-tested in early 2025, in accordance with corresponding guidelines [32]. Nucleic acid extraction and purification were performed as previously described. Each sample was tested in parallel using the AP Biotech kit and RealStar^®^ Dengue RT-PCR Kit 3.0 (Altona Diagnostics, Hamburg, Germany), as a comparator assay. The clinical cutoff was established by receiver operating characteristics (ROC) curve analysis. The optimal cutoff was selected according to the maximum Youden’s index, and the area under the ROC curve (AUC) was calculated to assess overall diagnostic accuracy. Qualitative agreement was assessed using 2 × 2 contingency tables. Clinical sensitivity and specificity were calculated relative to the comparator assay, along with total agreement. 95% confidence intervals were estimated using the Wilson score method, and concordance between assays was evaluated using Cohen’s kappa coefficient. Clinical performance was considered excellent when sensitivity, specificity, and total agreement were ≥95% [32] and the kappa coefficient was between 0.81–1.00 [33].

Clinical performance was further evaluated against an additional comparator method in an independent laboratory. One hundred additional residual serum samples (50 DENV-positive and 50 DENV-negative), collected from febrile patients during the 2023/2024 and 2024/2025 dengue outbreak seasons by the Centro de Tecnología en Salud Pública, Universidad Nacional de Rosario, were included. Prior to inclusion, samples had been characterized using the Centre for Disease Control and Prevention (CDC) DENV1–4 Real-Time RT-PCR Assay (Cat. No. KK0129) [34]. This assay was used as a multiplex real-time RT-PCR method for the qualitative detection and differentiation of DENV-1, DENV-2, DENV-3, and DENV-4.

All samples were extracted and tested with the evaluated kit as described above. In cases of discordant results, resolution testing was performed using RealStar^®^ Dengue RT-PCR Kit 3.0 (Altona Diagnostics, Hamburg, Germany). Clinical sensitivity, specificity, 95% confidence intervals, and kappa agreement were calculated as previously described.

#### 2.5.2. Serum–Plasma Equivalence

An additional analysis was conducted to assess equivalence between serum and plasma specimens. Ninety-five paired samples obtained from the same patients were analyzed using the evaluated kit. In case of discordant results, paired samples were further tested with an additional method, RealStar^®^ Dengue RT-PCR Kit 3.0 (Altona Diagnostics). Qualitative agreement was assessed as described above. For the comparison of Ct values, the mean paired difference, standard deviation and standard error of the mean difference and corresponding 95% confidence interval were calculated using a paired-samples *t*-test in MedCalc software version 23.4.2 [33].

## 3. Results

### 3.1. Analytical Performance

#### 3.1.1. Analytical Sensitivity

**Figure 1:**
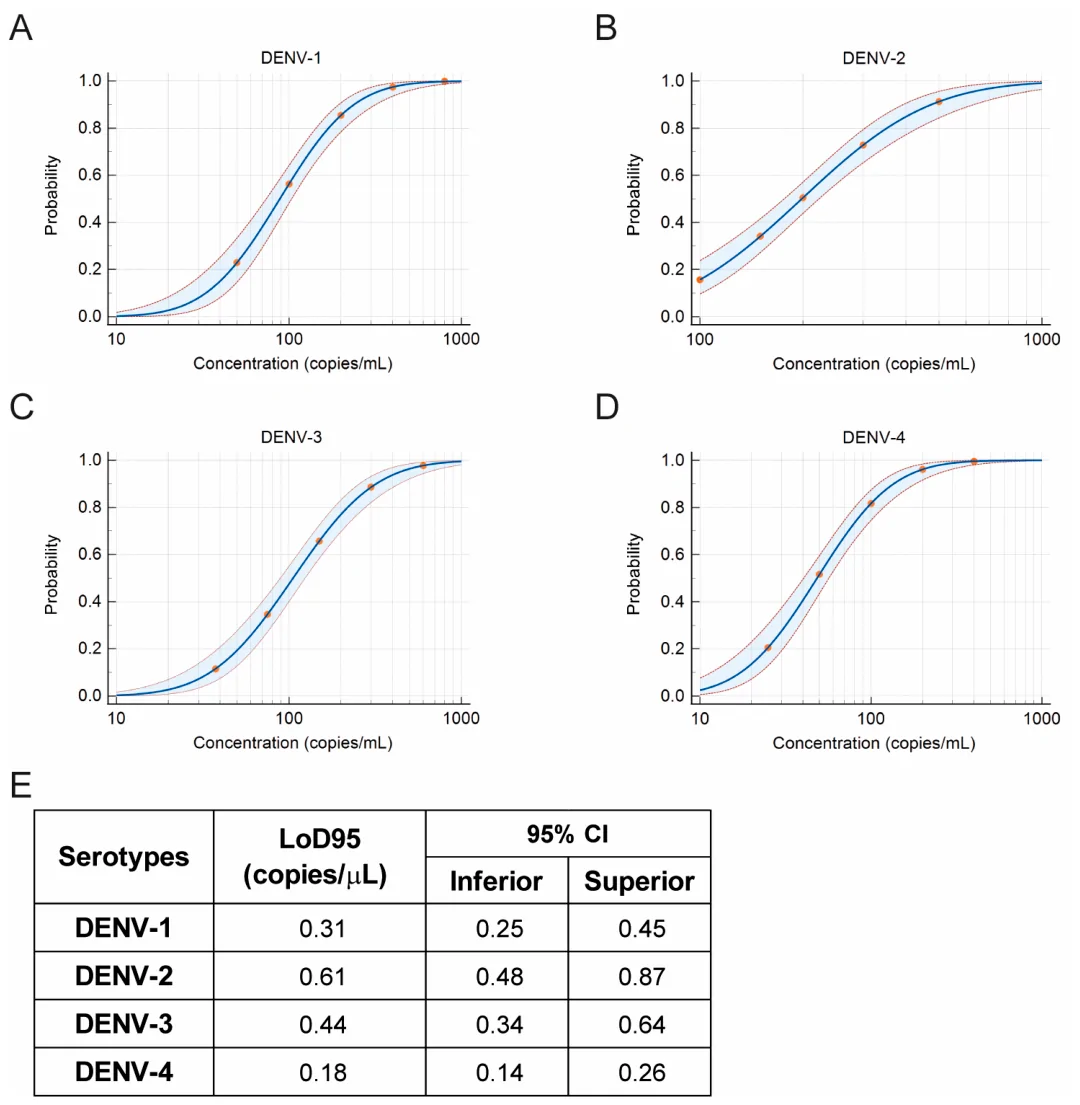
Analytical Sensitivity for DENV Serotypes. (**A**–**D**) Limit of Detection at 95% probability (LoD_95_) for DENV-1 to DENV-4, estimated using Probit regression analysis. Dots represent the observed detection results at each tested viral RNA concentration. The solid curve represents the fitted detection probability estimated by Probit regression, while the light blue shaded area and orange dotted lines indicate the corresponding 95% confidence interval. Viral RNA concentrations are expressed in copies/mL. (**E**) The corresponding LoD_95_ value and its 95% confidence interval (CI) are summarized in the table, expressed in copies/µL.

Serotype-specific LoD95 values were calculated separately for each reagent lot using a Probit regression model (Figure 1A–E). Supplementary Table S1 summarizes the dilution-series results for DENV1–4, including mean Ct values, positivity rates, and hit rates at each concentration, indicating that DENV1–4 maintained high detection rates near their estimated LoD95 concentrations, with hit rates across the dilution series supporting consistent low-copy RNA detection for all four serotypes. To ensure a conservative estimate, the reported LoD95 for each serotype corresponds to the highest LoD value obtained between reagent lots (Figure 1E). The overall limit of detection for DENV-specific RNA ranged from 0.18 to 0.61 copies/µL, demonstrating robust and comparable analytical sensitivity across DENV1–4.

Additional assessment of serotype equivalent detection included PCR efficiency and linear response to analyte concentration of the AP Biotech Discovery^®^ Dengue Virus RT-PCR Detection Kit. The correlation between Ct values and analyte concentration (in logarithmic scale) for each dengue serotype demonstrated a linear relationship across the evaluated range (Supplementary Figure S1). Linear regression analysis confirmed high assay efficiency and linearity, with similar slopes among serotypes and high correlation coefficient (R^2^) values, indicating equivalent amplification performance across all four DENV serotypes.

#### 3.1.2. Analytical Specificity

To examine potential analytical interference, eight candidate substances commonly present in serum or plasma samples in patients with signs related to DENV disease were tested. Interference testing demonstrated that DENV detection was not affected by any of the evaluated endogenous or exogenous substances (Supplementary Table S2).

In low-positive DENV samples, all tested conditions remained detectable, with 100% positivity across interferents and no relevant Ct shifts in the FAM channel compared with matched controls. These results indicate that haemoglobin, bilirubin, triglycerides, cholesterol, serum proteins, acetaminophen, amoxicillin, and dexamethasone did not compromise DENV RNA detection at the evaluated concentrations. Internal control amplification was also preserved across all conditions, with 100% VIC positivity and no relevant Ct shifts, supporting the absence of amplification inhibition or sample-processing interference (Supplementary Table S3). Overall, the assay maintained reliable DENV detection in the presence of clinically relevant interfering substances.

Analytical specificity was further evaluated by in vitro cross-reactivity testing against 15 clinically relevant non-target microorganisms at medically relevant concentrations, including closely related or clinically overlapping arboviruses. No DENV signal was detected in any replicate for any of the tested pathogens, corresponding to 0/15 cross-reactive microorganisms and 100% negative agreement in the cross-reactivity panel (Supplementary Table S4).

#### 3.1.3. Analytical Precision

Analytical precision was assessed to estimate the variability observed under routine laboratory conditions, including within-run repeatability, day-to-day variability, and inter-operator variability. Table 1 exhibits the degree of agreement among repeated measurements of each sample and SD and CV calculated by ANOVA (Table 1).

All panel samples with concentrations above the LoD exhibited the expected positivity rates, while the negative sample yielded 98.67% negative results for DENV when at least a 95% negativity rate was expected; internal control (IC) replicates were 100% positive, as expected. The CV corresponding to DENV detection for within-run repeatability ranged from 0.55–2.62% across all panel samples. Additional sources of variability showed CV values between 0.00–6.06% for operators, 0.00–3.91% for days, and 0.00–4.39% for reagent lots. Across all concentration levels, the assay demonstrated consistent performance, with low variability and no significant loss of detection at the evaluated concentrations.

Percentage agreement and analysis of variance results further supporting assay interlaboratory reproducibility are summarized in Table 2. The CV corresponding to DENV detection for interlaboratory variability ranged from 1.07–3.92% across all panel samples. All CV% values were within the predefined acceptance criteria for intra-laboratory precision and interlaboratory reproducibility.

### 3.2. Clinical Performance

#### 3.2.1. Diagnostic Performance in Serum Samples

To evaluate the diagnostic performance of the assay in serum samples, a panel of 296 human serum specimens from clinically suspected dengue patients was analyzed in parallel using the AP Biotech Discovery^®^ Dengue Virus RT-PCR Detection Kit and the comparator method. ROC analysis was applied to establish the clinical cutoff for the evaluated method. A Ct threshold of 40 was selected as the optimal cutoff, corresponding to the maximum Youden’s index (0.978), where samples with Ct values below 40 were considered DENV-positive (Supplementary Figure S2). The ROC curve yielded an area under the curve (AUC) of 0.989 (*p* < 0.001). A total of 178 samples were identified as Positive for DENV RNA by both the comparator method and the AP Biotech multiplex real-time RT-qPCR assay, while four samples were classified as Negative by the AP Biotech assay and Positive by the comparator assay. Both assays determined 114 samples as Negative for DENV (Table 3). Accordingly, the clinical sensitivity was 97.80% (95% CI: 94.49–99.14%), and the clinical specificity was 100.00% (95% CI: 96.74–100.00%). Total agreement exceeded 98%, with a Cohen’s Kappa coefficient of 0.97, demonstrating excellent diagnostic performance in serum samples. All previously serotyped samples tested DENV-positive for both tests, further supporting detection of DENV1 and DENV2 in clinical samples. Clinical samples characterized as DENV-3 and DENV-4 were not available during the study period; therefore, detection of these serotypes was supported by analytical validation studies only.

Additional external verification of clinical performance in serum samples was conducted through a retrospective, blinded study with 100 serum samples characterized by the comparator method CDC DENV1-4 Real-Time RT-PCR Assay. In total 50 tested Positive for DENV and 46 Negative by both the evaluated and comparator assays. Four discordant samples initially classified as false positives by the AP Biotech assay were re-evaluated and confirmed as Positives using the additional comparator, consistent with the results obtained by the evaluated test (Table 4). The AP Biotech Discovery^®^ Dengue Virus RT-PCR Detection Kit demonstrated optimal diagnostic performance, achieving 100% sensitivity and specificity, 100% total agreement, and a kappa coefficient of 1.0, indicating perfect concordance with the reference method.

#### 3.2.2. Diagnostic Performance in Plasma Samples

An external clinical evaluation was conducted using 221 human plasma samples. The AP Biotech assay identified 113 samples as Positive and 107 as Negative for DENV, with only one discordant result compared to the comparator assay (Table 5). The assay achieved a clinical sensitivity of 100.00% (95% CI: 96.71–100.00%) and a clinical specificity of 99.07% (95% CI: 94.94–99.84%) (Table 5). Total agreement exceeded 99%, with Cohen’s Kappa coefficient of 0.99, confirming excellent diagnostic performance in plasma samples.

#### 3.2.3. Serum–Plasma Equivalency

A complementary analysis was performed to assess equivalency in DENV detection between 95 paired serum and plasma samples. Of these, 47 pairs tested DENV-Positive in both matrices, and 47 pairs tested Negative in both matrices, while one pair showed a discordant result (Positive in plasma and Negative in serum) (Table 6). The total agreement was 98.95%, with a Cohen’s kappa coefficient of 0.98. The discordant pair was further evaluated using an additional method, which confirmed the initial findings, yielding a positive result in plasma and a negative result in the corresponding serum sample. This isolated discrepancy may be attributable to matrix-related differences affecting viral RNA recovery or extraction efficiency. Overall, these findings support equivalent assay performance for DENV detection in serum and plasma samples.

A paired comparison of Ct values obtained for the DENV detection channel in serum and plasma samples is shown in Supplementary Figure S3. Similar detection levels were observed between both sample types, with mean Ct values of 28.86 for serum and 28.74 for plasma. No significant differences were found, adding further evidence of equivalent performance of the evaluated assay across both types of clinical samples.

## 4. Discussion

Dengue virus infection continues to pose a substantial and increasing global health burden. In recent years, environmental changes have contributed to widening the incidence to areas not previously affected [35]. Accurate and timely molecular diagnosis is critical during the viremic phase, when viral RNA is detectable and serological markers are still absent. Real-time RT-PCR is the reference method for early DENV diagnosis due to its superior analytical sensitivity and specificity when compared to conventional RT-PCR or antigen-based assays. Real-time RT-PCR has proven reliable in detecting and differentiating all four serotypes (DENV1 through DENV4) across a broad range of viral loads in clinical samples, supporting both clinical management and epidemiological surveillance [34,36].

The AP Biotech Discovery^®^ Dengue Real-Time RT-PCR Detection Kit was developed to detect all clinically relevant DENV serotypes by targeting multiple genomic regions (NS5 for DENV1, E for DENV2/4, and M for DENV3), combining both conserved and serotype-specific elements to maximize analytical performance. Our results demonstrated high linearity across the studied range for all serotypes, with similar slopes and correlation coefficients, indicating comparable amplification efficiency and minimal target-dependent bias. The achieved LoD95 ranged from 0.18 to 0.61 copies/µL, consistent with the sensitivity reported for other validated RT-PCR platforms targeting DENV RNA, including those designed for simultaneous detection and typing of all four serotypes [35,36,37].

In the context of molecular diagnostics for dengue, the AP Biotech Discovery^®^ Dengue Virus RT-PCR Detection Kit demonstrated several operational characteristics that may enhance its suitability for routine laboratory implementation. The assay is configured in a ready-to-use format requiring the combination of only two components, thereby simplifying reaction setup and reducing hands-on time. Simplified workflows have been associated with decreased risk of pipetting errors and improved reproducibility, particularly in high-throughput or resource-limited settings [38,39]. In contrast, alternative approaches that rely on multiple reagents prepared in-house or sourced from different suppliers may increase operational complexity and variability between runs, potentially impacting consistency in diagnostic performance.

A relevant feature of the assay is the inclusion of a human endogenous internal control detected within the same amplification reaction. Internal controls are considered essential in nucleic acid amplification tests to monitor specimen quality, extraction efficiency, and the presence of PCR inhibitors [40]. The incorporation of a human endogenous internal control within the same amplification reaction enables simultaneous verification of specimen adequacy and proper sample processing without requiring additional reactions [41]. Compared with non-biological exogenous spike-in controls, endogenous targets more accurately reflect sample integrity and pre-analytical quality, thereby increasing confidence in negative results [42]. In our evaluations, the internal control signal remained stable even in samples with high viral RNA concentrations, suggesting limited susceptibility to competitive inhibition under conditions of elevated target load, as well as in the presence of potential interference from endogenous and exogenous substances.

Amplification curves were consistently interpretable, and no non-specific signals were observed during analytical testing. Additionally, the inclusion of formulated ready-to-use Positive Control as a component of the IVD kit allows for the confirmation of amplification setup, efficiency and reagent integrity, whereas Negative Control enables detection of contamination or non-specific amplification, thereby improving reliability and interpretability [43].

Another relevant operational characteristic of the AP Biotech Discovery^®^ Dengue Real-Time RT-PCR Detection Kit is the optimized thermal cycling program, which allows for completion of amplification in approximately 70 min. Reduced run times have important implications for laboratory efficiency, enabling increased daily testing capacity and improved instrument availability. Rapid molecular detection is particularly relevant during dengue outbreaks, as early laboratory confirmation facilitates appropriate clinical management and enables timely implementation of public health control measures [44]. Since the assay was validated as part of a diagnosis platform, involving semi-automated nucleic acid extraction and purification platform, the total turnaround time resulted in approximately 110 min from sample processing to result. Short turnaround times in molecular diagnostics have been associated with improved patient management and more efficient outbreak control strategies in viral infections [45].

Beyond its operational advantages and compared with other commercially available molecular assays, the AP Biotech Discovery^®^ Dengue Virus RT-PCR Detection Kit was subjected to comprehensive analytical and clinical validation in accordance with internationally recognized guidelines for in vitro diagnostic (IVD) devices. The validation included key parameters that are essential for establishing the reliability and diagnostic accuracy of molecular assays for dengue virus detection. Clinical validation was performed using both serotyped and non-serotyped DENV-positive clinical samples. Among the positive specimens, 56 had serotype characterization, including DENV-1 and DENV-2 samples, reflecting the serotypes circulating in Argentina during the study period. Although clinical samples identified as DENV-3 and DENV-4 were not available, the analytical validation demonstrated comparable assay performance across all four DENV serotypes.

Cross-reactivity assessment results confirm that the assay specifically detects DENV RNA without measurable cross-reactivity with other viral pathogens commonly associated with febrile illness or the differential diagnosis of dengue. Interference testing showed that common endogenous and exogenous substances did not affect the specific detection of DENV RNA, supporting reliable assay performance even in the presence of clinically relevant interferents. Overall, these findings demonstrate the absence of interference with dengue-specific detection and support the robust analytical specificity of the assay under the evaluated conditions.

Taken together, these performance characteristics suggest that the AP Biotech Discovery^®^ Dengue assay can reliably contribute to both clinical diagnosis and molecular surveillance efforts, facilitating evidence-based responses in endemic regions.

## 5. Conclusions

The AP Biotech Discovery^®^ Dengue Real-Time RT-PCR Detection Kit demonstrated high analytical sensitivity, balanced serotype detection, strong specificity, and excellent clinical concordance in both serum and plasma samples. Its robust and reproducible performance across DENV1 through DENV4 supports reliable early diagnosis during the acute viremic phase. In the context of expanding dengue transmission and increasing serotype co-circulation, these findings position the assay as a valuable tool for integrated diagnostic and epidemiological response in a rapidly evolving global dengue landscape.

## Figures and Tables

**Table 1:** Percent agreement results and analysis of variance for precision.

Sample	Average Ct	Agreement Rate *	Percentage Agreement (IC95%)	Within-Run		Day		Operator		Lot	
				SD	%CV	SD	%CV	SD	%CV	SD	%CV
FAM channel (DENV)											
Moderate Positive	35.25	148/150	98.67 (95.27–99.63)	0.92	2.62	1.17	3.33	1.23	3.48	0.79	2.25
Low Positive	36.46	147/150	98.00 (94.29–99.32)	0.89	2.44	1.43	3.91	2.21	6.06	1.60	4.39
Negative	N/A	148/150	98.67 (95.27–99.63)	N/A	N/A	N/A	N/A	N/A	N/A	N/A	N/A
VIC channel (Internal Control)											
Moderate Positive	31.52	150/150	100.00 (97.50–100.00)	0.36	1.15	0.00	0.00	0.12	0.38	0.14	0.45
Low Positive	31.38	150/150	100.00 (97.50–100.00)	0.17	0.55	0.00	0.00	0.00	0.00	0.00	0.00
Negative	31.30	150/150	100.00 (97.50–100.00)	0.18	0.59	0.00	0.00	0.24	0.76	0.27	0.86

* Agreement rate with the expected result; N/A: not applicable.

**Table 2:** Percent agreement results and analysis of variance for interlaboratory reproducibility.

Sample	Average Ct	Agreement Rate *	Percentage Agreement (IC95%)	Within-Run		Day		Interlaboratory	
				SD	%CV	SD	%CV	SD	%CV
FAM channel (DENV)									
Moderate Positive	35.06	75/75	100.00 (95.13–100.00)	0.44	1.26	0.93	2.65	1.07	3.05
Low Positive	36.18	75/75	100.00 (95.13–100.00)	0.63	1.76	1.25	3.46	1.41	3.92
Negative	N/A	75/75	100.00 (95.13–100.00)	N/A	N/A	N/A	N/A	N/A	N/A
VIC channel (Internal Control)									
Moderate Positive	31.58	75/75	100.00 (95.13–100.00)	0.21	0.66	0.18	0.58	0.28	0.88
Low Positive	31.42	75/75	100.00 (95.13–100.00)	0.17	0.55	0.17	0.55	0.17	0.54
Negative	31.27	75/75	100.00 (95.13–100.00)	0.20	0.65	0.24	0.76	0.26	0.83

* Agreement rate with the expected result. N/A: not applicable.

**Table 3:** Clinical performance of AP Biotech Discovery^®^ Dengue Virus RT-PCR Detection Kit in serum samples.

		Comparator Method		
		Positive	Negative	Total
AP Biotech Discovery^®^ Dengue Virus RT-PCR Detection Kit	Positive	178	0	178
	Negative	4	114	118
	Total	182	114	296
	Value	95% Confidence Interval		
Sensitivity	97.80%	94.49–99.14		
Specificity	100.00%	96.74–100.00		
Total agreement	98.65%			
Kappa	0.97			

**Table 4:** External verification of clinical performance in serum samples.

		Comparator Method		
		Positive	Negative	Total
AP Biotech Discovery^®^ Dengue Virus RT-PCR Detection Kit	Positive	50	0	50
	Negative	0	50	50
	Total	50	50	100
	Value	95% Confidence Interval		
Sensitivity	100.00%	92.87–100.00		
Specificity	100.00%	92.87–100.00		
Total agreement	100.00%			
Kappa	1.00			

**Table 5:** Clinical performance of AP Biotech Discovery^®^ Dengue Virus RT-PCR Detection Kit in plasma samples.

		Reference Method		
		Positive	Negative	Total
AP Biotech Discovery^®^ Dengue Virus RT-PCR Detection Kit	Positive	113	1	114
	Negative	0	107	107
	Total	113	108	221
	Value	95% Confidence Interval		
Sensitivity	100.00%	96.71–100.00		
Specificity	99.07%	94.94–99.84		
Total agreement	99.55%			
Kappa	0.99			

**Table 6:** Equivalency in DENV detection in serum and plasma samples.

		Serum		
		Positive	Negative	Total
Plasma	Positive	47	1	48
	Negative	0	47	47
	Total	47	48	95
	Value	95% Confidence Interval		
Positive Agreement	100.00%	92.44–100.00		
Negative Agreement	97.92%	89.10–99.63		

## Data Availability

The datasets generated and analyzed during the current study, as well as primers, probes, enzymes and other component information, are not publicly available because they contain proprietary information related to product design and development, which is protected under industrial confidentiality agreements. Data may be available from the corresponding author from AP Biotech upon reasonable request and subject to approval by the company’s confidentiality policies.

## Supplementary Materials

The following supporting information can be downloaded at: https://www.mdpi.com/article/10.3390/v18080914/s1, Figure S1: PCR efficiency calculation and linear response to analyte concentration; Figure S2: Clinical cutoff calculation; Figure S3: Serum–plasma Ct comparison. Table S1: Average Ct values and positivity rate for DENV1-4. Table S2: Interference testing in DENV detection. Table S3: Interference testing in internal control detection. Table S4: Analytical specificity analysis in samples with potential cross-reactive viruses.

## Abbreviations

The following abbreviations are used in this manuscript:

ANMAT	Administración Nacional de Medicamentos, Alimentos y Tecnología Médica
ANOVA	analysis of variance
ATCC	American Type Culture Collection
AUC	area under the curve
BDS	Guangzhou BDS Biological Technology Co., Ltd.
CDC	Centers for Disease Control and Prevention
CHIKV	Chikungunya virus
CI	confidence interval
CLSI	Clinical and Laboratory Standards Institute
CMV	Cytomegalovirus
Ct	cycle threshold
CV	coefficient of variation
DENV	Dengue virus
EBV	Epstein–Barr virus
eNCM	DENV-negative biological matrix
FAM	6-carboxyfluorescein
Flu A	Influenza A H1N109
Flu B	Influenza B
HBV	Hepatitis B virus
HCV	Hepatitis C virus
HIV-1	Human immunodeficiency virus type 1
HSV-1	Herpes simplex virus type 1
HSV-2	Herpes simplex virus type 2
IC	internal control
IS	interfering substance
IVD	in vitro diagnostic medical device
LoD	limit of detection
NAAT	nucleic acid amplification technology
OLS	ordinary least squares
PCR	polymerase chain reaction
PFU	plaque-forming unit
qPCR	quantitative PCR
ROC	receiver operating characteristic
RT-PCR	reverse transcription polymerase chain reaction
SD	standard deviation
SLEV	St. Louis encephalitis virus
UDG	uracil-DNA glycosylase
VIC	proprietary fluorescent dye used for internal-control detection
VZV	Varicella-zoster virus
WNV	West Nile virus
YFV	Yellow fever virus
ZIKV	Zika virus
