# Supplementary material for: Clinical and Analytical Performance of the AP Biotech Discovery® Dengue Virus RT-PCR Detection Kit
Source: Viruses. 2026 Aug 20;18(8):914. doi: 10.3390/v18080914 (PMC13517321; doi:10.3390/v18080914)
Supplement: Supplementary file 1 [file viruses-18-00914-s001.zip › viruses-4495593-supplementary.pdf]

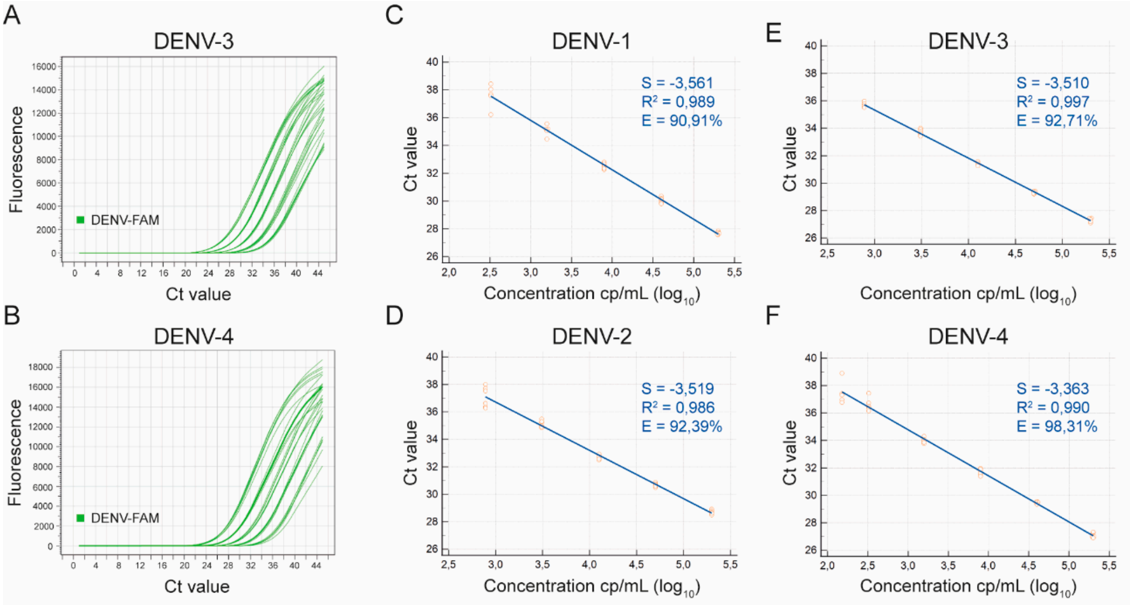

Figure S1. PCR efficiency calculation and linear response to analyte concentration. PCR amplification was performed using a QuantGene 9600 system (Bioer). Representative amplification curves are shown for DENV-3 (A) and DENV-4 (B). Linear regression analysis is shown for Ct values obtained for different analyte concentrations (RNA copies/mL in logarithmic units), for DENV-1 (C), DENV-2 (D), DENV-3 (E), and DENV-4 (F). The slope of the standard curve (S), the coefficients of determination (R^2) and the corresponding PCR amplification efficiencies (E) shown for each serotype indicate consistent and reliable amplification performance across the tested concentration range.

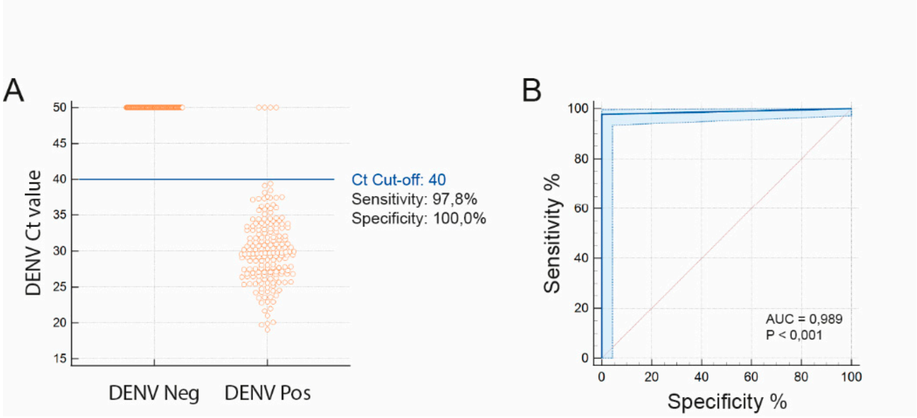

Figure S2. Clinical cutoff calculation. Receiver operating characteristic (ROC) analysis was performed to establish the clinical cutoff for serum samples. (A) Distribution of DENV Ct values obtained for DENV-negative (n = 114) and DENV-positive (n = 182) samples. A Ct value of 50 was assigned to negative samples for ROC analysis. The cutoff value was set at 40 to obtain sensitivity and specificity of 97.8% and 100.0%, respectively. (B) The area under the ROC curve (AUC) was 0.989 ($p < 0.001$), demonstrating excellent discriminatory capacity.

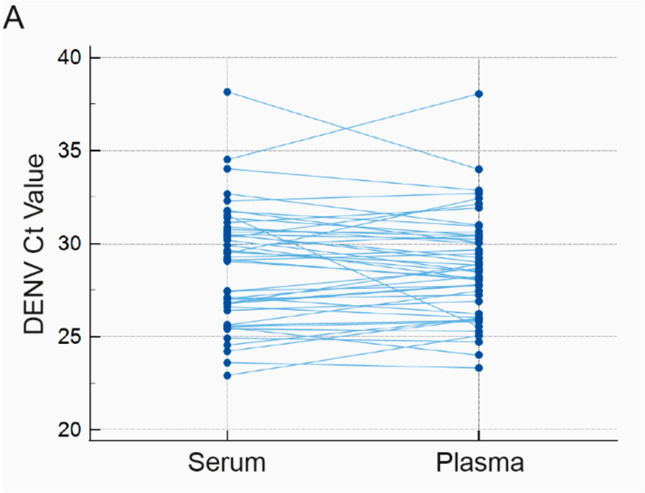

Figure S3. Serum–Plasma Ct comparison. (A) Paired comparison of Ct values obtained from serum and plasma samples for DENV detection. A total of 47 DENV-positive pairs (serum and plasma samples) were analyzed. A paired t-test showed no significant difference between the two sample types (P value = 0.619; mean difference = -0.122; 95% confidence interval: -0.368 to 0.611).

Table S1. Average Ct values and Positivity rate for DENV1-4.

DENV RNA Concentration (copies/mL)	Lot 1			Lot 2		
	Average Ct	Positive Rate (%)	Hit rate	Average Ct	Positive Rate (%)	Hit rate
DENV-1						
800	35.69	100%	54/54	36.08	100%	54/54
400	36.82	98%	53/54	36.88	98%	53/54
200	37.98	87%	47/54	38.38	85%	46/54
100	39.09	65%	35/54	39.73	52%	28/54
50	41.28	30%	16/54	41.68	26%	14/54
DENV-2						
500	37.96	96%	52/54	38.00	96%	52/54
300	38.76	78%	42/54	39.26	65%	35/54
200	39.42	59%	32/54	39.74	52%	28/54
150	40.30	35%	19/54	40.76	30%	14/54
100	40.74	30%	16/54	41.06	20%	11/54
DENV-3						
600	36.58	100%	54/54	36.84	100%	54/54
300	37.99	87%	47/54	38.02	89%	48/54
150	38.87	70%	38/54	39.56	61%	33/54
75	40.48	28%	15/54	40.69	30%	16/54
37,5	41.39	17%	9/54	41.38	17%	9/54
DENV-4						
400	36.10	100%	54/54	36.41	100%	54/54
200	37.25	98%	53/54	37.24	98%	53/54
100	37.85	80%	43/54	37.97	85%	46/54
50	38.78	44%	24/54	38.73	46%	25/54
25	38.89	26%	14/54	39.31	31%	17/54

Table S2. Interference testing in DENV detection.

FAM channel (DENV)				
**Interfering substances**	**DENV+**			
	**Control Av. Ct**	**Tested Av. Ct**	**Pos. Rate**	**%I**
Bilirubin	35.56	35.65	15/15	0.26%
Human Hemoglobin	35.56	35.60	15/15	0.12%
Human Serum Proteins	35.56	36.05	15/15	1.39%
Triglycerides	35.56	35.58	15/15	0.05%
Cholesterol	36.15	36.04	15/15	-0.32%
Acetaminophen	36.67	35.83	15/15	-0,13%
Amoxicillin	36.67	35.53	15/15	-0,97%
Dexamethasone	36.67	35.18	15/15	-1,92%

Table S3. Interference testing in Internal Control detection.

VIC Channel (IC)									
**Interfering substance**	**Concentration**	**DENV -**				**DENV+**			
		**Control Av. Ct**	**Tested Av. Ct**	**Pos. Rate**	**%I**	**Control Av. Ct**	**Tested Av. Ct**	**Pos. Rate**	**%I**
Bilirubin	475 µmol/L	28.26	28.20	15/15	-0.20%	28.22	28.18	15/15	-0.15%
Human Hemoglobin	2 g/L	28.26	28.23	15/15	-0.10%	28.22	28.26	15/15	0.13%
Human Serum Proteins	1 g/L	28.26	28.97	15/15	2.53%	28.22	28.91	15/15	2.45%
Triglycerides	37 mmol/L	28.26	28.18	15/15	-0.28%	28.22	28.08	15/15	-0.51%
Cholesterol	3 mmol/L	28.65	28.97	15/15	1.12%	28.62	29.09	15/15	1.64%
Acetaminophen	100 µg/mL	28.46	28.32	15/15	-0.49%	28.22	28.26	15/15	0.16%
Amoxicillin	7.5 mg/L	28.46	28.55	15/15	0.29%	28.22	28.50	15/15	0.99%
Dexamethasone	5 µg/mL	28.46	28.60	15/15	0.50%	28.22	28.59	15/15	1.31%

Table S4. Analytical specificity analysis in samples with potential cross-reactive viruses.

**Viruses**	**Concentration (copies/mL)**	**Positivity rate**
Chikungunya virus (CHIKV)	$1x10^5$	0/3
Cytomegalovirus (CMV)	$1x10^5$	0/3
Epstein–Barr Virus (EBV)	$1x10^5$	0/3
Hepatitis B Virus (HBV)	$1x10^5$	0/3
Hepatitis C Virus (HCV)	$1x10^5$	0/3
Herpes Simplex Virus type 1 (HSV-1)	$1x10^5$	0/3
Herpes Simplex Virus type 2 (HSV-2)	$5x10^4$	0/3

Human Immunodeficiency Virus type 1 (HIV-1)	$1x10^4$	0/3
Influenza A Virus H1N109 (Flu A)	$1x10^5$	0/3
Influenza B Virus (Flu B)	$1x10^5$	0/3
St. Louis Encephalitis Virus (SLEV)	$1x10^5$	0/3
Varicella-Zoster Virus (VZV)	$2x10^3$	0/3
West Nile Virus (WNV)	$1x10^5$	0/3
Yellow Fever Virus (YFV)	$1x10^5$	0/3
Zika Virus (ZIKV)	$1x10^5$	0/3
